# The Family and Pregnancy Pop‐Up Village: Developing a one‐stop shop of services to reduce pregnancy care‐related inequities in San Francisco

**DOI:** 10.1111/birt.12839

**Published:** 2024-06-17

**Authors:** Malini A. Nijagal, Osamuedeme J. Odiase, April J. Bell, Alison M. El Ayadi, Schyneida Williams, Chloe Nicolaisen, Garrett Jacobs, Brandi Mack, Monique LaSerre, Chelsea Stewart, KaSelah Crockett, Patience A. Afulani

**Affiliations:** ^1^ Department of Obstetrics, Gynecology & Reproductive Sciences University of California San Francisco California USA; ^2^ Institute for Global Health Sciences University of California San Francisco California USA; ^3^ Department of Family and Community Medicine University of California San Francisco California USA; ^4^ Department of Epidemiology & Biostatistics University of California San Francisco California USA; ^5^ Department of Obstetrics, Gynecology and Women's Health Albert Einstein College of Medicine/Montefiore Medical Center New York New York USA; ^6^ Designing Justice + Designing Spaces (DJDS) Oakland California USA; ^7^ Rafiki Coalition for Health and Wellness San Francisco California USA; ^8^ KaCierge Consulting, Compass & Keys Oakland California USA

**Keywords:** birth equity, community–institution partnership, health equity, perinatal health, PRECEDE‐PROCEED model, pregnancy, program planning and evaluation

## Abstract

**Introduction:**

Centering affected individuals and forming equitable institutional–community partnerships are necessary to meaningfully transform care delivery systems. We describe our use of the PRECEDE‐PROCEED framework to design, plan, and implement a novel care delivery system to address perinatal inequities in San Francisco.

**Methods:**

Community engagement (PRECEDE phases 1–2) informed the “Pregnancy Village” prototype, which would unite key organizations to deliver valuable services alongside one another, as a recurring “one‐stop‐shop” community‐based event, delivered in an uplifting, celebratory, and healing environment. Semi‐structured interviews with key partners identified participation facilitators and barriers (PRECEDE phases 3–4) and findings informed our implementation roadmap. We measured feasibility through the number of events successfully produced and attended, and organizational engagement through meeting attendance and surveys.

**Results:**

The goals of Pregnancy Village resonated with key partners. Most organizations identified resource constraints and other participation barriers; all committed to the requested 12‐month pilot. During its first year, 10 pilot events were held with consistent organizational participation and high provider engagement.

**Conclusion:**

Through deep engagement and equitable partnerships between community and institutional stakeholders, novel systems of care delivery can be implemented to better meet comprehensive community needs.

## Graphical abstract

The Family & Pregnancy Pop‐Up Village: Developing a one‐stop‐shop of services to reduce pregnancy care‐related inequities in San Francisco.

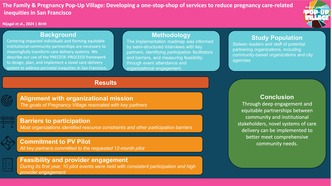

## 1 INTRODUCTION

Systemic, institutional, and interpersonal racism are root causes of disparate pregnancy care outcomes in the United States.^1^, ^2^, ^3^, ^4^, ^5^, ^6^, ^7^ For communities facing these inequities, achieving optimal outcomes requires comprehensive services beyond what is traditionally delivered through healthcare systems, such as support accessing health insurance, housing, healthy food, and other necessities. However, accessing available services is often prohibitively burdensome.^5^, ^6^ Urban areas like San Francisco have healthcare, public health, and “wraparound” services delivered in silos, making access difficult for those with limited job security, childcare, and transportation. Additionally, pregnant people of color experience racism and discrimination while receiving clinical and government services, posing yet another barrier to access.^4^ These barriers are particularly problematic in pregnancy due to frequent touchpoints, and because stress is associated with poor outcomes.^8^, ^9^ To achieve perinatal health equity, new models to reduce barriers and improve experience must be designed in partnership with those with lived experience, who best understand the problem.

Oriented by this goal, our team previously engaged in a 1‐year human‐centered design (HCD) process centered on pregnant Medicaid‐insured people in San Francisco, with a focus on those experiencing the deepest inequities: Black‐identifying individuals.^10^ During the *Inspiration* and *Ideation* phases, we discovered three foundational needs: easier access to services, better interactions with providers, and institutional efforts to earn trust of historically harmed communities. Celebrating pregnancy rather than pathologizing it was also emphasized, highlighting a need to shift toward treating pregnancy not as a medical problem but as a significant life transition that necessitates equal attention to social, emotional, and practical factors as is given to medical factors. During the *Brainstorming* and *Prototyping* phases, the concept of “Pregnancy Village” emerged—a community‐based, community–institutional co‐led model of providing social, public health, clinical, and wraparound services as a one‐stop shop, in an intentionally designed, celebratory, and uplifting environment.^10^ This model disrupts traditionally siloed systems in which groups work independently of one another; addresses structural inequities surrounding access; and facilitates coordinated care delivery between cross‐sector organizations. Importantly, the model explicitly focuses on continuous community engagement and responsiveness to meet community's changing needs.

In this paper, we describe the process of moving the Pregnancy Village concept to fruition and the feasibility and organizational engagement during the implementation pilot year.

## 2 METHODS

**FIGURE 1:**
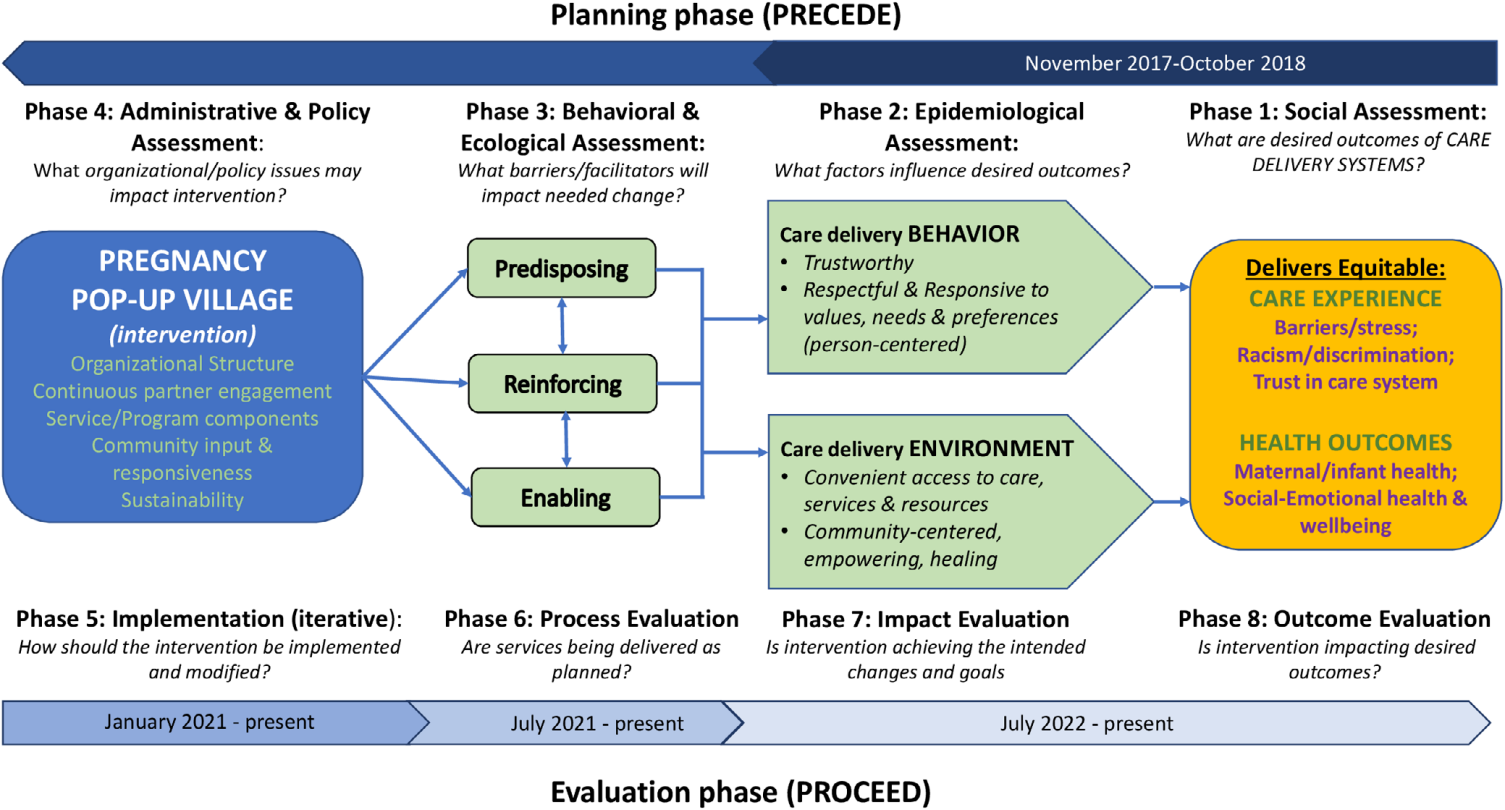
The PRECEDE‐PROCEED model for PV planning, implementation, and evaluation. [Colour figure can be viewed at wileyonlinelibrary.com]

The PRECEDE‐PROCEED framework guided the Pregnancy Village (PV) design, implementation, and evaluation efforts.^11^ This framework has traditionally focused on producing individual health behavior change. However, recognizing the influence of care delivery systems—structures, processes, and interpersonal interactions—on pregnancy care outcomes, our application of PRECEDE‐PROCEED focuses on driving behavior change in the *systems* that create inequities, rather than the individuals that experience them. PRECEDE phases 1 (understanding desired outcomes) and 2 (identifying factors that influence these outcomes) were completed through our previously published HCD work, and key learnings were used to create a logic model (Figure 1).^10^ As described in that publication, the work was co‐led by an academia‐based physician (MN) and a community‐based organization (CBO)‐based community health worker (SW) and was overseen by an advisory board comprised of institutional and community partners.

### 2.1 PRECEDE phases 3 and 4: Behavioral, ecological, administrative, and policy assessment

In phases 3 and 4, we engaged six key organizations or programs to assess factors that might affect their participation. These six (Table 1) were identified during phases 1 and 2 as being most frequently accessed and valued by our focus population; the goal was that all six would commit to delivering services at every event (“core vendors”), thereby ensuring the value of PV's one‐stop‐shop model. Of note, healthcare delivery systems (clinics and hospitals) were also identified as key stakeholders; however, the logistical and operational complexities of delivering clinical services with privacy, continuity, and nonrepetition precluded their inclusion in the pilot phase. From July to December 2020, we conducted 11 formal, semi‐structured interviews with 16 leaders and staff of the potential partnering organizations. Interviews focused on identifying existing gaps between their organizational goals and current service delivery, perspectives on the potential value of PV, and facilitators and barriers to delivering services within the proposed model. Each interview was conducted by members of the implementation team (CN and MN) and began by introducing the foundational needs identified during phases 1 and 2 and how PV might address them. Interviews were either recorded with permission or detailed field notes were taken. Although information that could be used to identify individual participants was accessible, the data were de‐identified before analysis.

**TABLE 1:** Health and wellness co‐anchor planning meeting attendance (January–June 2022).

Key organization or program (Partners)	Organization type	Key Services	Date of Attendance—Planning meetings and events											
			Jan‐22		Feb‐22		Mar‐22		Apr‐22		May‐22		Jun‐22	
			Meeting	Event	Meeting	Event	Meeting	Event	Meeting	Event	Meeting	Event	Meeting	Event
Homeless Prenatal Program^a^	CBO	CBO focusing on pregnant people and families with children 0–5 with services including connection to basic needs, classes, housing, legal services, employment opportunities, etc.			X	X	X	X		X	X	X	X	X
San Francisco Human Service Agency (HSA)^a^	City agency	Enrollment in Medicaid, food (SNAP) and cash assistance (TANF) programs, childcare, and more	X	X	X	X	X	X	X	X	X	X	X	X
Black Infant Health (BIH)^a^	City Agency	state‐funded program that provides culturally affirming services, including prenatal or postpartum group sessions, individual life planning sessions, breastfeeding classes, nurse support, and other community activities	X		X	X	X	X	X					
Home Visiting Program^a^	City Agency	State‐funded program that provides a home visiting nurse during pregnancy and up to 2 years postpartum, providing information about what to expect during pregnancy and childbirth, baby care and development, and more	X	X	X	X	X	X	X	X	X	X	X	X
Women, Infant & Children's (WIC) Program^a^	City Agency	State‐funded nutrition program that provides nutrition, education, breastfeeding support, and a debit card to purchase healthy foods at the grocery store	X	X	X	X	X	X		X	X	X		X
SisterWeb Doula Organization^a^	CBO	CBO provides Black, Latine, and Pacific Islander pregnant persons and their families a free birth doula with expertise, support, and advocacy to promote a satisfying and dignified birth experience	X	X	X	X	X	X	X	X	X	X	X	X
Rafiki Coalition for Health & Wellness	CBO	Mental health services, therapeutic messages, chiropractic services, navigation services, food resources, and connection to other community resources	X	X	X	X	X	X	X	X	X	X	X	X

*Note*: We started consistently collecting organizations' attendance at planning meetings and events in January 2022.

^a^ Six key stakeholder groups were identified during Phases 1 and 2 as being most frequently accessed and valued by pregnant people of color and/or those living on low incomes. Three of these groups were programs of the San Francisco Department of Public Health.

### 2.2 PROCEED Phase 5: Implementation

Phase 5 began with exploring optimal processes and leadership structures for achieving the deeper goals of PV: building trust with community and creating a celebratory and uplifting care delivery environment. Three key areas were explored:

1. *Forming a leadership team and structure*: Phases 1 and 2 revealed that institutional partners' humility, new perspectives, and willingness to change are necessary to build trust between communities of color and institutions or systems of care. Co‐leadership between institutional‐based and community‐based leaders is thus critical, along with a structure fostering trust building and transparency, bi‐directional learning, and equitable power sharing and decision‐making. To assemble the leadership team, the academia‐based SF Respective Initiative (SFRi), which led the initial HCD work, sought out local, mission‐aligned CBOs to co‐lead the process of further designing and implementation planning. CBOs were identified based on SFRi team members' community relationships, recommendations of trusted colleagues, and assessments of CBO expertise and/or community positioning that would be necessary for model implementation. This process started immediately after the PV concept was solidified in November 2018.
2. *Creating a shared vision and approach among participating organizations* (*core vendors*): Deep engagement among core vendors around PV's vision and foundational goals was considered essential for success. Planning meetings with these groups began in January 2021. Before the first meeting, a survey containing four open‐ended questions was sent out to the leaders and on‐the‐ground liaisons for the six groups that committed to becoming core vendors, as well as Rafiki coalition, a CBO that would play both leadership and core vendor (service delivery) roles for PV (*n* = 16). The survey was designed to spark transparent conversations about partners' ambitions and concerns around the PV effort and perspectives on making participation valuable and sustainable. Planning meetings were held every 6 to 8 weeks initially and monthly once events launched. Meetings lasted 90 minutes and were held using video conference, with one in‐person meeting held at the planned event site.
3. *Building processes to sustain core vendor engagement and participation*: Learnings from phases 3–5 were consolidated to develop concrete processes for engaging core vendors outside events. These processes focused on promoting continued participation, alignment between PV and vendors' goals, communication around conflicts or barriers, and bi‐directional learning toward achieving the shared vision.

### 2.3 PROCEED Phase 6: Process evaluation

During the first year, the process evaluation assessed model feasibility and engagement of the six initial core vendors. Visitor attendance and experience at PV events were also assessed and will be presented separately. Measures of feasibility included capacity to deliver monthly events over 1 year and core vendors' attendance to provide the “one‐stop shop” of services. Organizational engagement was measured by attendance at monthly planning meetings and bi‐monthly assessments of the partner engagement process (PEP), using a modified version of the Expecting Justice Framework.^12^ The Expecting Justice Framework allows project organizers to assess partners' level of engagement and receive feedback on meeting agendas, activities, and communications.^12^ The 15 PEP items employed a 5‐point Likert‐type response ranging from strongly agree to strongly disagree, and responses to the PEP scale were summed to generate a total score (range 0–60), where low PEP scores indicated poor perceptions of the partner engagement process. Scores were then generated for its four subscales: (1) openness and trust; (2) meeting effectiveness and progress; (3) use of data to inform strategy; and (4) comfort talking about racism (Appendix S1).

#### 2.3.1 Analysis

To analyze the qualitative interviews, we used rapid analysis, an applied strategy for obtaining actionable, targeted qualitative data in a quicker timeframe than standard qualitative methodologies, and for assessing important intervention elements, facilitators, and barriers.^13^ We first developed a template to summarize interview data.^14^ The interview guide was used to create this template, which included a priori key themes based on program objectives. Interview data were summarized under each theme, and representative quotes were selected.^14^ During analysis, the template and summary were updated with emergent themes identified from the data. We conducted descriptive analysis of quantitative data. For provider engagement surveys, we generated summative scores from the full PEP and subscale items, standardized for score comparability (range 0–100).

## 3 RESULTS

### 3.1 PRECEDE Phases 3 and 4: Behavioral, ecological, administrative, and policy assessment

Interviews revealed a clear commitment by all six groups to become core vendors. Factors that affect sustainable implementation are summarized by themes, with representative quotes presented in Table 2.

**TABLE 2:** Selected quotes from core vendors for qualitative themes and sub‐themes.

Theme	Organization type	Quote
*Organizational service delivery goals*	City‐led group or org	“Making sure that our participants are getting adequate care, equitable care and they're not being discriminated upon […] better care coordination, the ability to really be transparent and honest and open about the availability of what we can do, or what we cannot do, as service providers”
*Existing gaps between organizational goals and current practices*	City‐led group or org	“Staffing can (also) be impacted in some kind of way by all of the hoops and hurdles that have to happen for us to be able gain access to all of the things that we need”
	City‐led group or org	“All the things that we're sharing with our moms, that are going to happen, don't happen! There's kind of poor follow through or no ability to really meet the needs that are desired by the families that we're sharing with them are available”
	CBO	“It's one thing to refer somebody somewhere and then they get on the waitlist with somebody and have to wait. We're not really being able to see them through to success. I would really like to see our partnerships forge more direct access for our clients”
*Potential Impact of PV*	CBO	“[There is] a lot of the extra stuff that our doulas need to do right now ‐ that's been increased because of the pandemic, whether it's getting signed up for other services or access to healthy food, or getting your questions answered around legal matters. The doulas are showing up and doing it because it's what needs to be done. I feel like if there were a space where a lot of these other service agencies or care providers were going to be there, instead of running town and trying to help her client get housing, she could say, “Oh, there is going to be a housing clinic tent here. Why don't we show up together,” and “it's more of a warm handoff instead of the doula having to do stuff outside her area of expertise”
	CBO	“They [Homeless pregnant persons] don't knock on our door first; they go to the coordinated entry and the coordinated entry sends them to us. But they don't send them to us soon enough. We would much prefer to have women early on in their pregnancy. Our first woman who moved in had a baby already. I mean, come on, this (getting transitional housing) is really supposed to help women have a peaceful time during their pregnancy where they can take care of themselves, take care of their unborn child, deliver a healthy baby, get prepared for it, make sure that they're getting the care and support they need during this time in their life”
	CBO	“A beautiful space. That's what people need. They need to feel valued. When there is something that is made for them that is nice, that works, that they have input into, it's important”
*Facilitators to participating in PV*	City‐led group or org CBO	“They (Black clients) have been impacted in a negative way…that is not just traumatizing to the population we're serving, but it's also traumatizing to the staff and employees who are passionate about doing the work and getting it done…(the) vision I see behind the Pregnancy Village: I think it is amazing the vision of it” “If we had a person who could help organize and delegate all of our community engagements and connections, then I think we'll be a lot more effective in how we use people's time and how we do say yes or no to things that are actually going to progress us towards our goals”
*Barriers to participating in PV*	City‐led group or org	“The other thing … we have been thinking about, is like, how can we provide the services… (like height and weight) …when we may not have all the tools available right there to do it… I don't know”
	City‐led group or org CBO	“It is a bureaucratic program, I'm not going to lie about it. Its goal, yes, is to help people, but a lot of the policies revolve around fraud prevention and that's what like is ‐ sometimes seems to be the focus that there isn't a very holistic approach to delivering services” “We have more than enough staff to get out the door and give to people, but we don't really have the staff available to [send to PV events] … but I think even without staff, it's an important thing to be part of. It's an important project to be able to lend what you do have”
*Organizational needs to facilitate and sustain PV participation*	City‐led group or org	“I think it's amazing the vision of (PV), but I do think that some of the agencies have to be accountable for the areas in which they're lacking. They have to improve in that before sitting at the table, because they just further traumatize it, and it makes it seem like we're all on board with it. That is the one think that is very concerning to me”
	City‐led group or org	“Trying to look at if we are reaching out to more Black or Hispanic moms that are coming in through the area, because … we don't have much presence in that community, just because the clinic that we currently have there is only open once a month”

1. *Organizational service delivery goals*: Vendors emphasized the goal of “meeting clients where they are” to ensure optimal access. Providing respectful care, building trust with families through empathy and transparency, provision of coordinated care, meeting the needs of community, and the responsibility of care systems to evolve and transform were noted as critical goals for optimal service delivery.
2. *Existing gaps between organizational goals and current practices*: Interviewees identified multiple factors stifling their ability to meet clients' needs, including suboptimal access, a lack of organizational capacity, limited staffing, insufficient coordination with other organizations, and bureaucratic policies and administrative burdens, including extensive documentation requirements and inadequate systems to support efficient and effective communication. The inability to meet clients' needs, along with a lack of capacity for follow‐up due to limited staffing, were noted to inhibit trust building with clients.
3. *Potential Impact*: Interviewees highlighted that by providing opportunities for streamlined referrals and warm hand‐offs to other partners on‐site, PV could improve efficiency, reduce redundancies, and allow each group more time to provide direct services. Bringing traditionally harder‐to‐access programs into PV could result in earlier intervention, such as getting pregnant, unhoused clients into transitional housing before birth. They also noted that PV might help them build trust with clients by showing they were “doing business differently” by bringing services to communities instead of requiring travel to their centers and by coordinating with other organizations; and being able to engage with clients in an uplifting and empowering space was conducive to trust building.
4. *Facilitators to participation*: Organizational leaders stated that PV's mission, oriented around antiracism and eliminating disparities among Black people, aligned with their organization's goals, and facilitated their participation. One leader highlighted that PV could improve staff satisfaction by allowing community‐based service delivery in a way that could be fulfilling. On‐the‐ground staff interviews confirmed enthusiasm about participating in PV events. Providers expressed the desire to be valued for their knowledge and experience, which the PV setting might better allow for. Some noted that onsite childcare services and food could facilitate their participation, given that events would occur on Saturdays.
5. *Barriers to participation*: Multiple organizations highlighted chronic underfunding, overworked staff, and the impact of COVID‐19 on organizational staffing as significant barriers to participating in PV. Program policies and requirements were also mentioned as a potential barrier. For instance, one city‐led group noted that the highly structured nature of their state‐funded program made it difficult to tailor their services to different settings and wondered if the PV environment (setting, availability of technology and equipment, etc.) could accommodate requirements.^15^ Other state and federally funded programs mentioned various program rules (e.g., documentation for service initiation) that might limit their ability to meet clients' needs on site. However, they also noted that the COVID‐19 Public Health Emergency declaration (January 2020) had introduced more flexibility around rules or requirements, thereby mitigating this challenge in the short term. Finally, it was noted that PV's weekend schedule could be a barrier for employees who usually have a weekday‐only schedule; respondents believed this could be acceptable with appropriate compensation.
6. *Organizational needs to facilitate and sustain PV participation*: Some organizations requested more clarity around core partner expectations (i.e., number of staff needed at events, resource investment, etc.). Solid communication with PV leaders and fostering strong on‐the‐ground partnerships were identified as keys to ensuring PV success. Importantly, it was noted that, owing to historical harm and a lack of follow‐through for Black clients, trust building between participating organizations and between PV and the community would be critical to accomplishing PV's vision.

### 3.2 PROCEED Phase 5: Implementation

**FIGURE 2:**
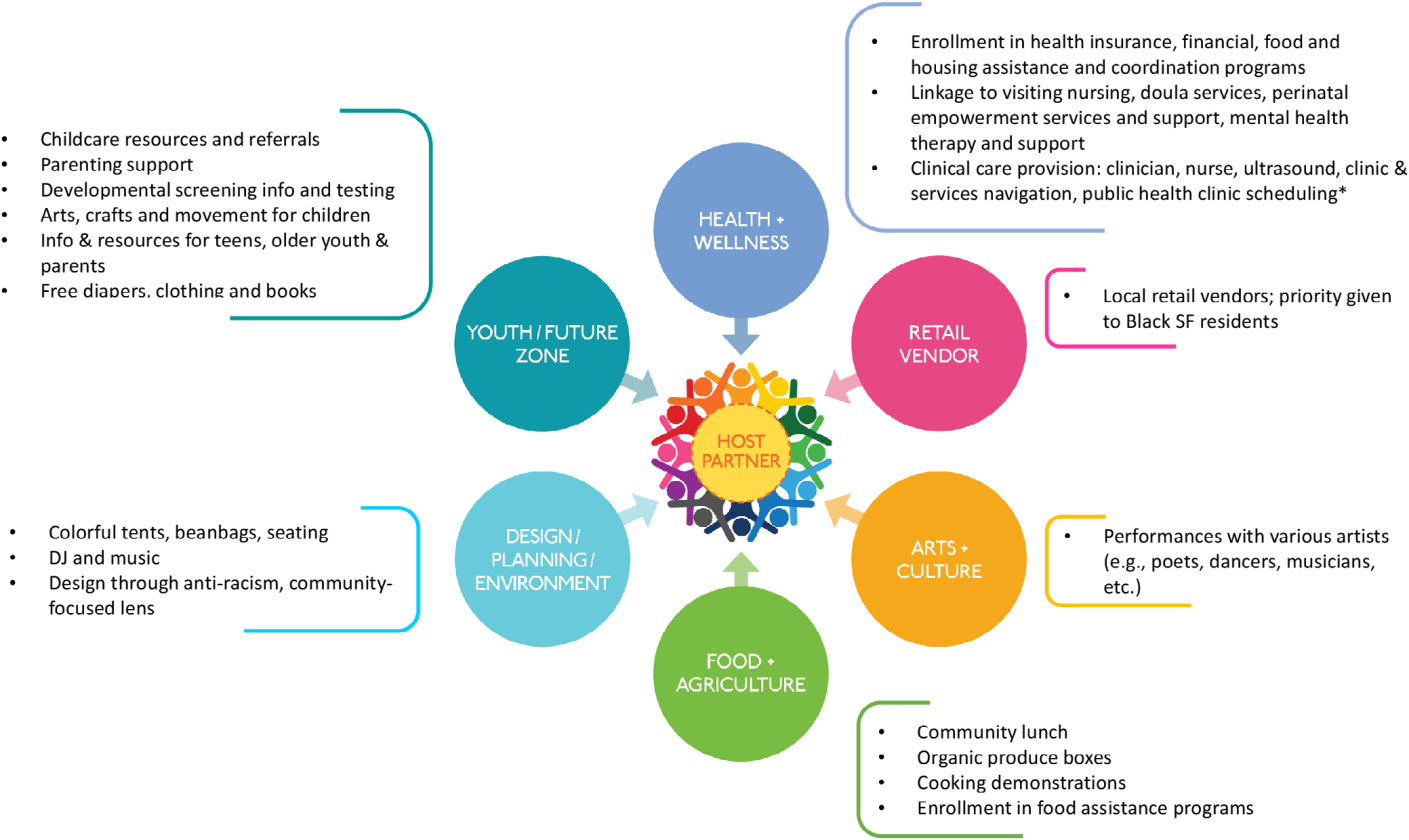
Family and Pregnancy Pop‐Up Village neighborhood hubs and summary of services provided. [Colour figure can be viewed at wileyonlinelibrary.com]

1. *Leadership team and structure*: SFRi partnered with two CBOs to form the initial leadership group that would launch the new model. The first was Designing Justice + Designing Spaces (DJDS), a Black‐led, nonprofit architecture and design firm that builds place‐based solutions addressing poverty, racism, unequal access to resources, and other structural inequities, with strong expertise in community engagement and co‐design. DJDS had developed the “Pop‐Up Village” (2017), a site activation project that unites a constellation of programming—health and wellness, youth and family, retail, food, and education resources—in customized buses, pop‐up shops, furniture, and other types of mobile architecture, with a mission to create a village of civic resources for underresourced communities so they can thrive.^13^ Given strong overlap in vision and goals, the Pop‐Up Village model was an optimal vehicle for implementing the Pregnancy Village vision, and SFRi and DJDS decided to launch a Family and Pregnancy‐focused Pop‐Up Village (FPPV) together in San Francisco (2019). DJDS would play a lead role in community‐engaged co‐design and event design, production, and operations. The second partner, Rafiki Coalition, joined in Spring 2020. A Black‐led, 35‐year‐old San Francisco CBO with a mission to improve the health and wellness of Black and other marginalized communities, Rafiki would engage the local community around FPPV and participate both as an organizer and a service provider (core vendor).
  DJDS' Pop‐Up Village model and organizational structure were developed to include six different areas (neighborhoods) of services, representing what is needed to support comprehensive wellness within communities (Figure 2). Each neighborhood would be led by 1–2 “anchor partner(s)”: organizations that would recruit vendors with specific skills, expertise, and/or services to participate in FPPV events. Ideally, each neighborhood would have an institutional anchor and a CBO anchor to leverage the different assets needed for community connectedness, partnership, and sustainability. The FPPV leadership team would be comprised of the anchor partners (current and future), host partner (providing the site for events), and program manager. Rafiki and SFRi would co‐anchor the Health & Wellness (H&W) neighborhood and DJDS the Design/Planning/Environment neighborhood.
  Before the FPPV event launch (July 2021), meetings between anchor partners occurred as needed. By January 2022, two monthly anchor partner meetings were implemented: one for the H&W co‐anchors and the other for the full leadership team. By June 2022, FPPV had solidified anchor partners for the Youth Zone and the Arts & Culture neighborhood.
  Anchor partner meetings included transparent conversations about factors essential for effective co‐leadership to meet collective goals. These included investing in one‐on‐one relationships between partners to build trust; being flexible and adopting an adaptive leadership model that would prioritize equitable partnerships between CBOs and city or institutional anchors; being able to stay true to each organization's own goals while also serving the collective goals; and being transparent and equitable with funding and fundraising. Challenges discussed included time conflicts that precluded partners from attending meetings where decision‐making occurred; conflicts between individual organizational and FPPV demands; differing bandwidth or ability to fundraise; reaching consensus around logistical processes; and balancing attention between event planning and the work to achieve FPPV's deeper goals.
2. *Creating a shared understanding, vision, and approach among H&W vendors*: Core vendor survey respondents expressed much enthusiasm about the new model, highlighting the potential for FPPV to be a supportive, antiracist, and responsive space that could truly center on clients' needs and excitement about interacting with community and each other in this new way. Respondents pointed out multiple concerns around the process, including: organizations have been functioning within a White supremacy culture and may not recognize if continued patterns of racism play out within FPPV; community members need to be included in all decisions, small and large; and commitment by all organizations to learn from each other and community is fundamental. Survey findings were shared at the first group meeting to facilitate discussion and understanding about how different partners were coming into the work and to set the stage for collaboratively developing community agreements, success measures, and other partnership processes.
3. *Building processes to sustain H&W core vendor engagement and participation*: Based on learnings from preimplementation interviews and the core vendor survey, several processes were established to facilitate FPPV goals, sustain partner participation, and ensure continued goal alignment. These included:
  - Clear articulation of commitment: Core vendors were asked to provide a staff lead to be the primary FPPV liaison, attending each event to deliver services and attending monthly planning meetings. Events occur on Saturdays and require a 6 to 7‐hour time commitment. Core vendors are not paid for their participation. Rather, FPPV events benefit organizations by providing infrastructure for community‐based service delivery in a novel environment, allowing for cross‐sector collaboration and trust building with community.
  - Monthly meetings with FPPV liaisons: Co‐anchors facilitated monthly H&W neighborhood meetings focused on three goals: (1) community building between vendors; (2) logistical planning for events; and (3) advancing FPPV's antiracism agenda. The third goal includes discussions on how to iterate in response to community feedback, where structural racism may be affecting FPPV's goals (i.e., lack of transportation to events), sustainable financing, and other areas. Within the first year, the group created community agreements, helped modify the PEP scale as a tool to self‐assess their engagement and engagement processes, developed a mission statement, and collaboratively problem‐solved around issues that arose at events. Of note, an additional monthly meeting, led by the FPPV project manager, was held for vendors across all FPPV neighborhoods to collaborate around logistical aspects of events and other village‐wide issues.
  - Communication processes with core vendors: Notes were distributed after each meeting to update those who could not attend. Online systems (Google Forms) were utilized by vendors to communicate resource needs that would be required for events. H&W co‐anchors communicated with core vendor leadership every 3–6 months by means of email or one‐on‐one meetings to give updates and elicit any feedback.
  - Assessment of partner experience: Core vendor experience, including opportunities for open‐ended feedback, was assessed every two meetings (see *Methods*: *Phase 6*). Co‐anchors monitored survey results to adjust the meeting structure as needed.
  - Transparency around community feedback from events: Community feedback from each event through vision boards and other engagement tools was made accessible to all partners and the community at large using a publicly available Google Document.

### 3.3 PROCEED Phase 6: Process evaluation

Feasibility: 10 of 11 planned pilot events were held, with one canceled due to a major COVID‐19 exposure among operations staff. Except for one city‐based group whose small staff was heavily affected by COVID‐19, all core vendors consistently attended FPPV events (Table 1). By the end of the first year, a total of 16 core vendors were participating in FPPV events. New partners that joined were community‐based, aligned with FPPV's mission, and provided services explicitly requested by the community by means of feedback mechanisms at events; examples included groups providing fatherhood support, education around perinatal mental health, and mobile dental services.

Partner engagement: Planning meetings were consistently attended, with each of the original core vendors attending at least 50% of meetings (Table 1). PEP surveys revealed high satisfaction with the partner engagement process (Table 3). Of the 38 responses recorded, the standardized PEP score was 93.3 (SD = 7.2).

**TABLE 3:** Distribution of standardized full partner engagement process and subscale scores.

Name of scale	*N*	Score	SD	Minimum Score	Maximum Score
Full partner engagement process	38	93.3	7.2	63.3	100.0
Openness and trust	38	95.8	7.8	62.5	100.0
Meeting effectiveness and progress	38	93.8	8.8	65.0	100.0
Comfort to speak about race or racism	38	95.4	8.9	62.5	100.0
Use of data to inform strategies	38	82.6	16.8	50.0	100.0

## 4 DISCUSSION

To our knowledge, our work represents the first application of PRECEDE‐PROCEED to create change in systems of care delivery, rather than in affected individuals, to achieve desired outcomes.^16^, ^17^, ^18^ Given extensive evidence of the role of systemic and institutional racism in creating disparate health outcomes, achieving health equity requires identifying problematic features within existing care delivery systems and designing alternative approaches. Deep collaboration between communities and institutions is essential to this process; those with lived experiences hold key insight into existing problems and potential solutions, and institutions are well positioned to move systems toward these solutions and create sustainable structural change. Insights from community members and care organizations during the PRECEDE phases allowed us to successfully design, implement, and conduct early evaluation of our novel model of care (PROCEED phases). This methodology as well as the community–institutional collaboration processes and resulting learnings we have described may be useful to other communities seeking to tackle health inequities at a systems level.

Our implementation of SF's Family and Pregnancy Pop‐Up Village demonstrates the feasibility of bringing cross‐sector organizations together around a unified vision based on antiracism principles. The model disrupts siloed systems that disproportionately burden people facing structural barriers by delivering services as a one‐stop‐shop, building in mechanisms for community feedback, and creating processes for responsive change. It acknowledges that communities of color may feel unsafe in historically harmful, built environments and creates an alternative dignified and uplifting care environment. FPPV's community–institutional co‐leadership model is also disruptive of usual innovation efforts. For example, although service co‐location is a common approach to improving healthcare efficiency and collaboration, such programs are generally embedded within one lead organization.^19^ The FPPV's leadership structure prioritizes the needs of the focus population over any one organization by having decision‐making shared between multiple committed stakeholder organizations. This collaborative leadership structure is critical given that trust building and deep learning between communities and institutions emerged as foundational needs. Nevertheless, few examples of such leadership models in health and social service delivery exist; we anticipate that lessons learned from the ongoing development and implementation of FPPV's leadership model will result in concrete and replicable process recommendations for creating equitable community–institutional partnership models.

Interviews revealed that strong alignment with FPPV goals was key to the participation of stakeholder organizations. Almost every organization stated financial and human resource limitations as potential barriers to participation; nevertheless, they were drawn to the novel opportunity to deliver services in community and collaborate with other organizations and community members in real time to meet needs. The timing of the launch, which occurred as COVID‐19 restrictions were beginning to loosen, may have facilitated success, as organizations were eager to re‐engage with clients in person. Thus, FPPV may have filled an additional need based on this timing alone.

The multiple strengths of our work include providing a successful example of community–institutional collaboration focused on systems change.^20^ Second, our foundational care delivery goals and resulting solution prototype (FPPV) are likely generalizable to other perinatal care communities in the United States, particularly in urban areas. For example, recent HCD work done with Black birthing people in Michigan revealed a similar vision for an optimal pregnancy care experience, including models to address basic needs such as housing, nutrition, and safety; modified care to decrease barriers; improved access to additional care team members (e.g., community health workers and doulas); and service colocation to improve access.^21^ Furthermore, although we specifically focused on perinatal care, the needs identified in our HCD process and the care delivery behavior and environment to fulfill those needs are relevant across the life course. As such, the Pop‐Up model could be used to tackle other health inequities.

Limitations in our work include that our implementation process was heavily aided by organizations already being aligned with an antiracism mission and an urgency around local inequities in perinatal outcomes. In communities lacking the same awareness or urgency, getting organizations to dedicate their time and resources to such an effort may be more challenging. We were also fortunate to have a strong group of local leaders who were open to exploring a new type of partnership requiring more transparency, vulnerability, and power sharing than usual. The need for this type of “readiness” and alignment may present a barrier to other communities trying to implement a similar model.

### 4.1 Conclusions

This study demonstrates the use of an implementation science framework and human‐centered design methods to create systemic change around pregnancy care inequities in San Francisco. Our success in bringing together cross‐sector organizations to implement a novel system in response to community's stated needs, is a promising example of how cities might approach tackling systemic care inequities. Deep collaboration among community partners, CBOs, and government agencies, a shared vision around creating systems change, and clear processes to facilitate creating this change are critical to achieving health equity.

## CONFLICT OF INTEREST STATEMENT

The authors have declared that no competing interests exist.

## Supporting information


Appendix S1.

## Data Availability

The data that support the findings of this study are available on request from the corresponding author. The data are not publicly available due to privacy or ethical restrictions.
