# Supplementary material for: The Family and Pregnancy Pop‐Up Village: Developing a one‐stop shop of services to reduce pregnancy care‐related inequities in San Francisco
Source: Birth. 2024 Jun 17;52(1):66–77. doi: 10.1111/birt.12839 (PMC11829268; doi:10.1111/birt.12839)
Supplement: Supplementary file 1 — Appendix S1. [file BIRT-52-66-s001.pdf]

Appendix 1. Items from the 15-item PEP scale by domains

Sub-scale	Items
Openness and trust (6)	Individual perspective reflected in guiding goals<br>The PV team fosters a culture of openness and trust<br>Individual perspective reflected in PV team vision<br>Individual perspective valued in meetings<br>Comfort expressing opinion in front of PV team<br>Feeling that right people are at the table for planning and implementation of PV<br>*Sub-score range*: 0 - 24
Meeting effectiveness and progress (5)	Meeting an effective use of time<br>Satisfaction with progress made by the collective<br>Satisfaction with focus areas<br>Meeting increased knowledge of other partners' programs and services<br>Clear goals, community agreements, and objectives for PV meetings<br>*Sub-score range*: 0 - 20
Use of data to inform strategies (2)	Data deepened understanding of how collective can address pregnancy care inequities<br>Common agenda informed by data<br>*Sub-score range*: 0 - 8
Comfort/ability to speak about racism (2)	Comfort discussing issues on race and racism among PV team<br>Understand how race and racism are related to pregnancy care inequities<br>*Sub-score range*: 0 - 8
